# Salmagundi

**Published:** 1887-02

**Authors:** 


### Salmagundi.

EDITED BY E. G. BETTY, D.D.S.

A bill to regulate the practice of dentistry and to repeal existing dental laws was introduced in the Indiana Senate on the 6th of last month.

The Aztec of to-day differs but little from that of Cortez time. He possesses a large mouth, white, short, regular teeth, which he seldom loses in old age.

The query on p. 47 of the last number, first line of Salma gundi, should have followed the clipping from *Science*, on p. 48, but the printer got so muddled that he didn't know just *where to land.*

A loaf of bread from a bakery, upon being cut open, revealed a set of teeth, presumably those of the baker. It is needless to say that loaf was not eaten, though well prepared for mastication.

The Mississippi Valley Association will be in session before the next issue of the Register reaches our readers, and we wish to remind them that one and all are welcome and expected to be present. Each one coming should be prepared to have something to do, say, or read, so that all will be mutually benefitted. The Register as usual will have a reporter present.

After Marriage, What?—A Cheyenne clergyman, the Rev. R. E. Field, lectures on the subject, "After Marriage, What?" Sometimes it's poverty, Mr. Field, occasionally divorce, but more frequently paregoric and soothing syrup, croup, mumps, and measles. Indeed, there are ever so many things after marriage—numerous old maids, for instance.—*Chicago Mail.*

DEATH records kept by the Philadelphia *Ledger* for the last fifteen years, of persons living to or beyond the age of eighty years, show an increase of from 659 in the year 1872, to 883 in the year 1886. In all years women largely outnumber men, in some years nearly two to one.—*Clipping.*

---

CAULK'S DENTAL ANNUAL for 1886–87 is at hand replete with statistics of great variety and usefulness. This issue is a double number of about 100 pp. and published at 50 cts., a price that is ridiculously small in comparison with the quantity and quality of matter between the covers. No dentist who wishes to be conversant with the *personel* of society officers, colleges, periodicals, etc., can afford to be without this dental—"Spofford's Almanac."

---

WASHINGTON was very fond of a certain brand of pickled tripe, put up in Bristol, England. In ordering some from the dealers, Cary & Co., London, he wrote: "Dear Cary, Mrs. Washington joins me in warm thanks to you for your considerate present of two large stone jars of pickled tripe. I must ask you to arrange for four similar jars to be shipped for my account. * * * * Dental infirmity impels my caring for this necessary item in our domestic commissariat."

---

HE SAW HE WAS FROM CHICAGO.—A well-known dentist contributes the following: A gentleman from the boundless West was calling at the "parlors" for consultation, and as the interview was about terminating his eye chanced to fall upon the doctor's diploma, the leading line of which, in very black text, ran thus: "Academia Chirurgia Dentium, etc., etc.

As the Chirurgia struck him his face suddenly lighted up, and, extending his hand energetically, he exclaimed: "Why, doctor, I didn't know that you were from Chicago. Shake!"—*From the Boston Record.*

---

SNOBBERY IN THE LAND OF SNOBS.—A curious little story was told me illustrative of the snobbishness of London society and of the real kindness of heart in the family of the Prince of Wales. There is in London an American dentist who is very skillful in his profession, and amongst other people has attended the Princess of Wales. He was invited to one of the annual garden parties at Marlborough House, and met there nearly fifty of his patients. They cut him to a man. Soon after he found himself face to face with the Prince and Princess, and they at once gave him the most cordial reception, and shook him warmly by the hand. At once every one of the fifty patients pressed up to the lucky dentist and shook hands with equal warmth.—*London Letter in the Sheffield Independent.*

---

Men of Mars who Quail before Tooth-tinkers.—Having occasion to visit a dentist the other day, I asked him if I was the most nervous man he ever had in this chair. "Bless you, no," replied the tooth-tinker. "You are only about the average patient, and it would surprise you to see how some of the big men act when they come here to get their teeth fixed. General Sheridan, for example, is one of the worst men in a dentist's chair you ever saw, and General Logan is even worse than he. Why, Logan is as tender as a baby, and will shrink at the slightest touch on his nerves, while I suppose, there were never two braver men in battle than they. General Sherman, too is about as bad, and acts as if he were going to have a fit whenever the drill is put into his teeth. He jumps, and grunts, and groans, and takes on worse than if he were having his leg cut off."—*Washington Letter in Chicago News.*

---

Electric Welding.—In electric welding, however, some of the metals which it was before impossible to weld become more easily dealt with; such as cast-iron, brass and bronze, zinc, tin, etc. Copper, formerly welded with so great difficulty and uncertainty, unites with great ease and certainty. Iron, steel, platinum and like metals, formerly known as weldable, are with great facility welded electrically. Thus far I have not tried any pieces of the same metal and failed to make a weld. When, however, the pieces are of different metals or alloys, failure may result from too great differences either in their temperature of softening or in their specific electrical and heat conductivities.

Before describing the details of my process, it may be briefly stated to consist in forcibly pressing together the bars or other pieces to be joined or welded and then passing an electric current of large volume through the pieces, a small portion of the bars on each side of the place of abutment serving as a path for the current. The resistance to the passage of the current at the meeting point of the bars gives rise to a welding heat at this point, and the pressure causes a thorough union, and generally a slight expansion at the union, caused by the approaeh of the pieces under the pressure. The uses are certainly not few, and an enumeration of some of them may be made. One of the most evident applications is in joining, end to end, wires of copper and iron for various purposes, such as in forming coils of magnets and in telegraph, telephone, and electric light lines for the avoidance of clumsy or resisting joints. * * * * *

There is also a very wide field of usefulness to be found in the manufacture and repair of tools and machinery.

As examples of such work I may mention the lengthening of screw-taps, drills, reamers, augers, to any desired degree; welding new drills and reamers to the taper shanks of old and worn-out drills and reamers; and in general welding steel pieces to steel or wrought-iron, or even cast-iron bodies of tools.

To make an electric weld, the pieces are rubbed bright near the ends to be joined, so as to make good contact with the clamps by which the current enters, they are then placed in the clamps with their ends abutted in the free space between the clamps. The ends are, of course clean, so as to form good contact, after which, in most cases, a little powdered borax is applied to the joint to act as a flux, or if the metal be of low melting point, as tin or lead, a little zinc chloride or tallow is applied.

It is, of course, best to have the clamps formed to fit the pieces, especially when they are of irregular outline, but for round or square work simple V grooves in the clamps suffice to hold the pieces in place and give the requisite contact.

When the pieces are in place and pressed together by the means provided, the current is turned on, and at once the ends of the bars heat at the junction, a slight yielding or approach of the pieces takes place, and before the operation could be described in words the work is done. Sometimes the joint is hammered to still further perfect and consolidate the weld.—*Prof. Thompson at Boston School of Technology.*

